# Leukocytic Toll-Like Receptor 2 Deficiency Preserves Cardiac Function And Reduces Fibrosis In Sustained Pressure Overload

**DOI:** 10.1038/s41598-017-09451-3

**Published:** 2017-08-23

**Authors:** Jiong-Wei Wang, Magda S. C. Fontes, Xiaoyuan Wang, Suet Yen Chong, Elise L. Kessler, Ya-Nan Zhang, Judith J. de Haan, Fatih Arslan, Saskia C. A. de Jager, Leo Timmers, Toon A. B. van Veen, Carolyn S. P. Lam, Dominique P. V. de Kleijn

**Affiliations:** 1 Department of Surgery, Yong Loo Lin School of Medicine, National University of Singapore, Singapore, Singapore; 2 Cardiovascular Research Institute (CVRI), National University Heart Centre Singapore (NUHCS) and National University Health System (NUHS), Singapore, Singapore; 3 Department of Medical Physiology, Division of Heart & Lungs, University Medical Center Utrecht, Utrecht, Utrecht, The Netherlands; 4 Laboratory of Experimental Cardiology, Department of Cardiology, Heart Lung Center Leiden, Leiden University Medical Center, Leiden, The Netherlands; 5 Department of Cardiology, University Medical Center Utrecht, Utrecht, The Netherlands; 6 Laboratory of Translational Immunology, University Medical Center Utrecht, Utrecht, The Netherlands; 7 National Heart Centre Singapore, Duke-NUS Graduate Medical School, Singapore, Singapore; 8 Cardiology, University Medical Center, Groningen, The Netherlands; 9 Department of Vascular Surgery, University Medical Center Utrecht, Utrecht, The Netherlands; 10 Netherlands Heart Institute, Utrecht, The Netherlands

## Abstract

An involement of Toll-like receptor 2 (TLR2) has been established in cardiac dysfunction after acute myocardial infarction; however, its role in chronic pressure overload is unclear. We sought to evaluate the role of TLR2 in cardiac hypertrophy, fibrosis and dysfunction in sustained pressure overload. We induced pressure overload via transverse aortic constriction (TAC) in TLR2^−/−^ and wild type (WT) mice, and followed temporal changes over 8 weeks. Despite similar increases in heart weight, left ventricular (LV) ejection fraction (EF) and diastolic function (mitral E/A ratio) were preserved in TLR2^−/−^ mice but impaired in WT mice following TAC. TAC produced less LV fibrosis in TLR2^−/−^ mice associated with lower mRNA levels of collagen genes (Col1a1 and Col3a1) and lower protein level of TGFbeta1, compared to WT mice. Following TAC, the influx of macrophages and CD3 T cells into LV was similar between TLR2^−/−^ and WT mice, whereas levels of cyto/chemokines were lower in the heart and plasma in TLR2^−/−^ mice. TLR2^−/−^ bone marrow-derived cells protected against LVEF decline and fibrosis following TAC. Our findings show that leukocytic TLR2 deficiency protects against LV dysfunction and fibrosis probably via a reduction in inflammatory signaling in sustained pressure overload.

## Introduction

Hypertension carries one of the highest population attributable risk factors for heart failure in the general population^1^. The development of left ventricular (LV) hypertrophy, diastolic dysfunction or reduction in ejection fraction signifies the presence of early Stage B heart failure in chronically hypertensive patients, and heralds progression to symptomatic Stage C heart failure^2^. Understanding the mechanisms underlying LV hypertrophy and dysfunction in sustained pressure overload is therefore critically important for the prevention of heart failure.

Inflammation is recognized as a key mechanism for heart failure progression. Circulating inflammatory markers, such as interleukin-6 (IL6) and tumor necrosis factor alpha (TNF-α) are elevated in patients with heart failure^3^. These markers are, among others, regulated by Toll-like receptors^4^–transmembrane receptors that recognize ‘pathogen-associated molecular patterns’ of exogenous microorganisms and ‘danger-associated molecular patterns’ of endogenous danger signals^5, 6^. We previously showed that Toll-like receptor 2 (TLR2) on leukocytes determined infarct size after ischemia/reperfusion injury and subsequent adverse LV remodeling^7, 8^. The role of TLR2 in LV hypertrophy and dysfunction without myocardial ischemia, however, is less clear.

Renal ischemia-induced LV hypertrophy was reduced in TLR2^−/−^ mice at 15 days after reperfusion, with a reduced systemic proinflammatory response^9^. In doxorubicin-induced cardiomyopathy without hypertrophy, TLR2 inhibition reduced LV ejection fraction (LVEF) decline and fibrosis at 8 weeks^10^. In contrast, angiotensin-induced LV hypertrophy was similar between TLR2^−/−^ and WT at 7 days, but TLR2 deficiency in bone marrow-derived cells reduced fibrosis and inflammation^11^. Data in transverse aortic constriction (TAC) models are conflicting: TLR2 deficiency exacerbated cardiac dysfunction in spite of reduced hypertrophy and fibrosis at 14 and 28 days in one study^12^; while in another study, TLR2 deficiency increased LV hypertrophy at the same time points^13^.

In this study, we induced sustained pressure overload using TAC for a much longer period of 8 weeks in TLR2^−/−^ and WT mice. We determined the temporal changes to identify the role of TLR2 in the development of LV hypertrophy, systolic/diastolic dysfunction, fibrosis and inflammation over time. We showed that TLR2 deficiency resulted in preservation of LV systolic/diastolic function and less LV fibrosis related to lower collagen formation and less cytokine/chemokine production in the TLR2^−/−^ heart. These changes were mediated by leukocytic TLR2, and not cardiac TLR2, in response to sustained pressure overload.

## Results

### TLR2 deficiency preserved cardiac function in hypertensive LV hypertrophy

**Figure 1:**
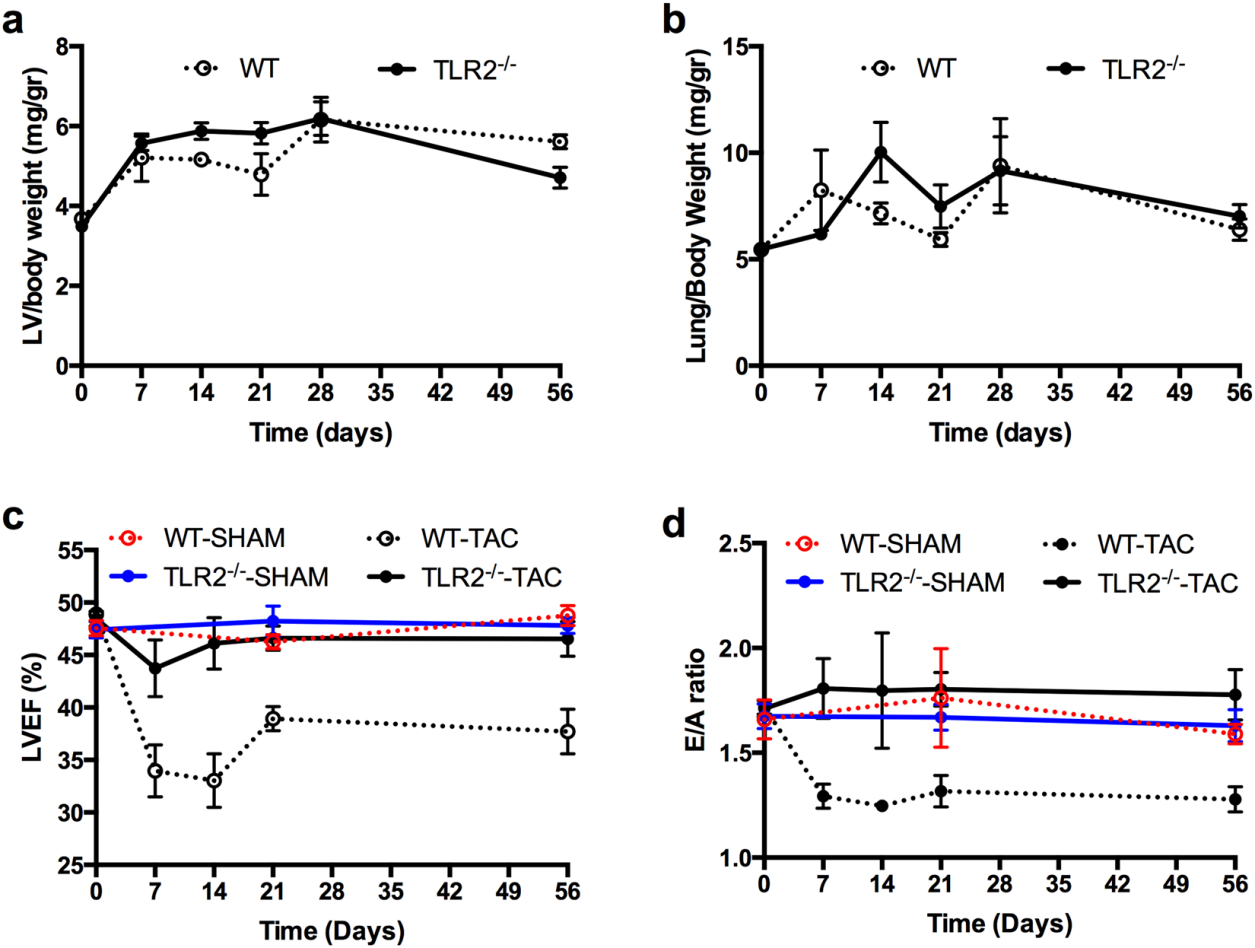
Left ventricle weight, lung weight and cardiac function after sustained pressure overload. (**a**,**b**) Mouse LV and lungs were extracted before TAC (baseline, n = 10–12 per genotype), or 7 days (n = 6–7 per genotype), 14 days (n = 8–10 per genotype), 21 days (n = 6 per genotype), 28 days (n = 6–9 per genotype) and 56 days (n = 21–26 per genotype) after TAC from WT and TLR2^−/−^ mice. Wet LV weight and lung weight were corrected for body weight. No significant differences in LV or lung weight between WT and TLR2^−/−^ mice were detected. (**c**,**d**) LVEF as an indicator for cardiac systolic function and E/A ratio as an indicator for cardiac diastolic function were determined with echocardiography at indicated timepoints after TAC. N = 14 for WT-SHAM, n = 12 for TLR2^−/−^-SHAM, n = 26 for WT-TAC and n = 22 for TLR2^−/−^-TAC. GLM model analysis was performed, *p* < 0.0001 between WT-TAC and TLR2^−/−^-TAC.

Sustained pressure overload was induced by TAC with less than 10% mortality in both WT and TLR2^−/−^ mice during 8 weeks follow-up. LV weights increased similarly following TAC in WT and TLR2^−/−^ mice (Fig. 1a) with all mice having a right/left carotid velocity ratio between 6 and 8. Also, mRNA levels of atrial natriuretic peptide (ANP) and brain natriuretic peptide (BNP), two hypertrophic markers, were increased similarly in both WT and TLR2^−/−^ mice (Supplementary Fig. S1). Lung weight did not differ between WT and TLR2^−/−^ mice (Fig. 1b). For cardiac function, LVEF was significantly lower in WT mice compared to TLR2^−/−^ mice during 8 weeks follow-up after TAC (Fig. 1c, *p* < 0.0001). Furthermore, E/A ratio was reduced in WT mice 7 days after TAC (*p* < 0.001 compared to Baseline, Fig. 1d), but not in TLR2^−/−^ mice, and was lower in the WT group compared to the TLR2^−/−^ group during 8 weeks follow-up after TAC (*p* < 0.0001). No significant change in LVEF or E/A ratio was observed in the sham-operated animals (Fig. 1c,d).

### TLR2 deficiency reduced interstitial fibrosis in the heart

**Figure 2:**
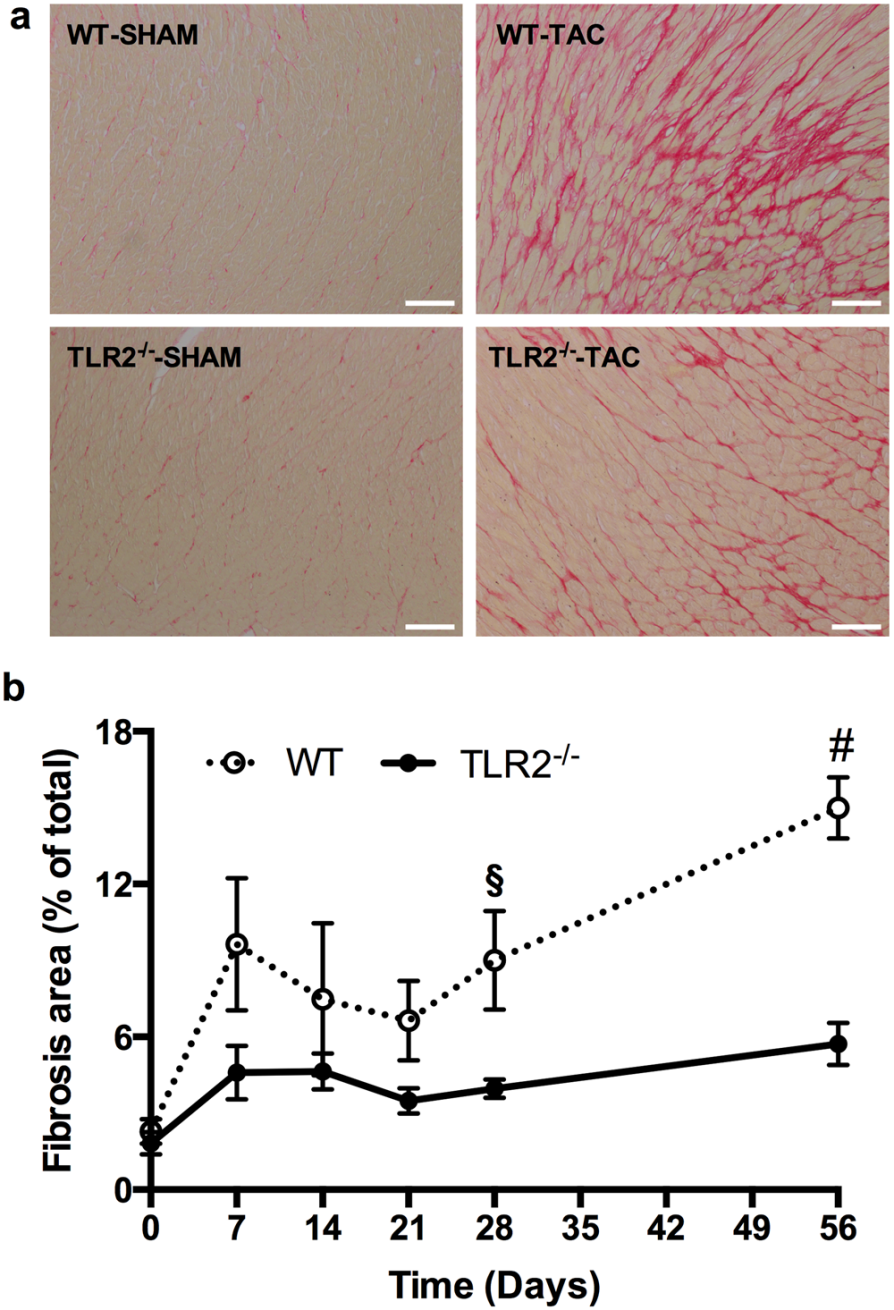
Interstitial fibrosis in the heart. (**a**) Representative images of heart sections stained with Picrosirius Red to show collagen deposition. Scale bars = 100 μm. (**b**) Quantification of Picrosirius Red stained area (fibrosis area) as % of the whole LV area at indicated timepoints after TAC. The numbers of mouse hearts extracted at each timepoint for each genotype of mice were the same as described in Figure 1. ^§^p < 0.01, ^#^p < 0.001.

Myocardial fibrosis is an important hallmark of maladaptive hypertrophy induced by pressure overload^14^ and is associated with myocardial stiffness and development of heart failure in hypertensive rats^15^. Having established that TLR2 deficiency protects against reduction of EF and E/A ratio following TAC, we investigated if interstitial fibrosis was lower in TLR2^−/−^ TAC hearts in accordance with a better cardiac function. As shown in Fig. 2a, severe interstitial fibrosis was observed in WT hearts after 8 weeks’ TAC but not in TLR2^−/−^ TAC hearts or sham hearts. Compared to baseline, cardiac fibrosis increased 7 days after TAC in both WT (*p* < 0.01) and TLR2^−/−^ (*p* < 0.05) mice. Comparing the WT-TAC with the TLR2^–/–^-TAC group during 8 weeks follow-up (Fig. 2b) showed that the TLR2^−/−^-TAC group had less interstitial fibrosis (p < 0.001). Cardiac fibrosis was significantly lower in TLR2^−/−^ mice compared to WT mice at 4 weeks (*p* = 0.002) and 8 weeks (*p* = 0.000).

**Figure 3:**
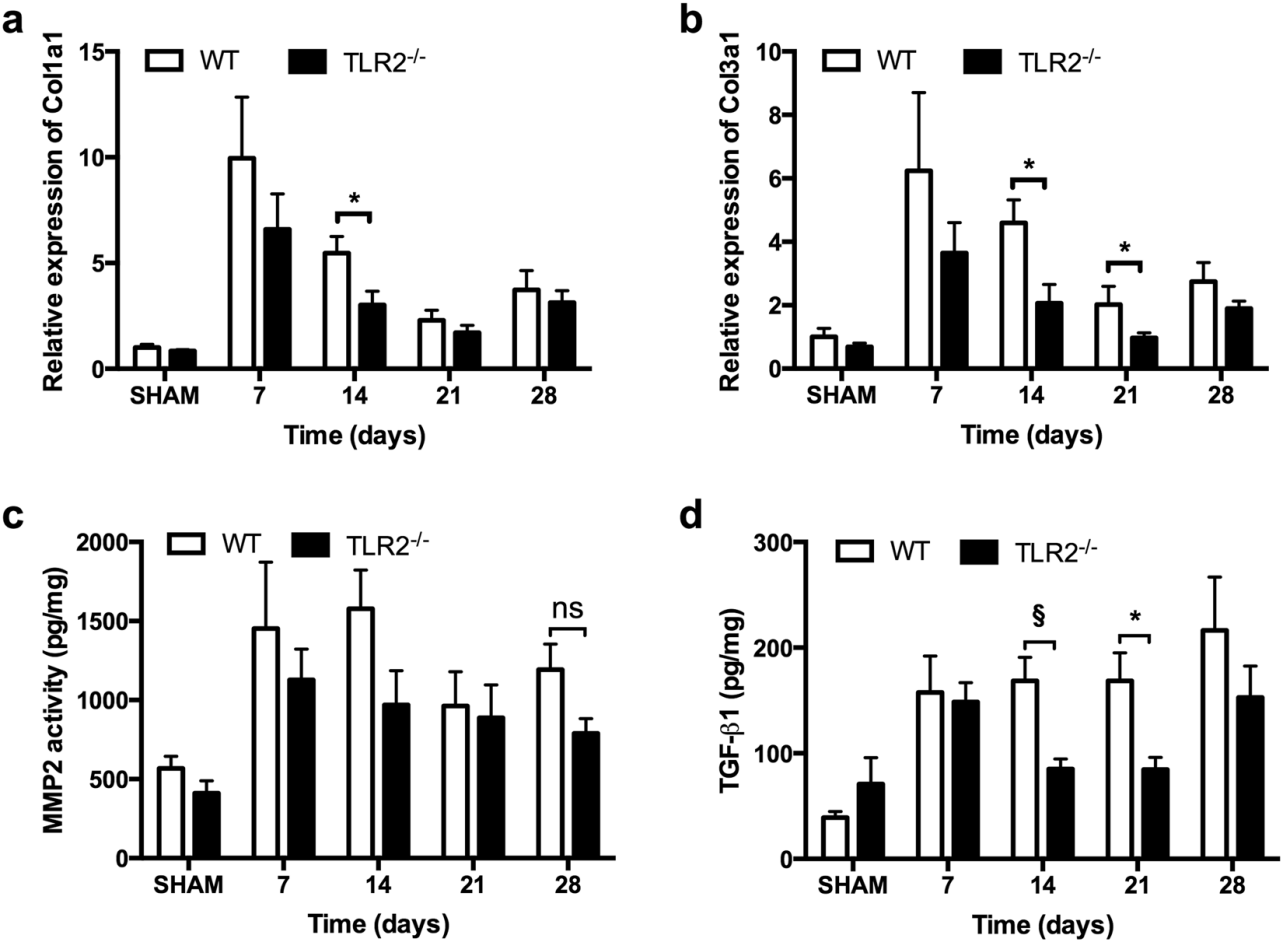
Collagen synthesis and breakdown in the heart. (**a**,**b**) Relative expression of Col1a1 and Col3a1 in the heart were quantified by qRT-PCR and normalized to WT-SHAM. GAPDH was used as an internal control. (**c**) Activity of MMP2 in heart tissue was determined at indicated timepoints. (**d**) Protein level of TGF-β1 in heart tissue was determined by multiplex assay at indicated timepoints. Bars represent mean ± SEM. Mouse hearts were extracted at indicated timepoints, n = 6–8 mice per genotype per timepoint. Mann Whitney U test was performed to determine the difference between groups at individual timepoints; *p < 0.05, ^§^ *p* < 0.01, ns indicating not significant.

To investigate if the reduced fibrosis in TLR2^−/−^ mice was due to reduced collagen production or increased collagen breakdown, mRNA levels of the most abundant cardiac Col1a1 and Col3a1 were determined as well as activity of the most abundant Matrix Metalloproteases (MMPs)-2 and -9. Messenger RNA levels of Col1a1 and Col3a1 were significantly lower in TLR2^−/−^ hearts compared to WT hearts after TAC (Fig. 3a,b, *p* < 0.05). MMP-2 and -9 activity levels, however, did not differ between TLR2^−/−^ and WT mice after TAC (Fig. 3c, Supplementary Fig. S2).

Transforming growth factor beta (TGF-β) is an important modulator of cardiac fibrosis with all three TGF-β isoforms (TGF-β1, TGF-β2 and TGF-β3) expressed in mammalian hearts^16^. Measurement of protein levels of TGF-β1, TGF-β2 and TGF-β3 in the heart after TAC showed that TGF-β1 levels in the heart were higher in WT mice compared to TLR2^−/−^ mice at 2 weeks (*p* = 0.006) and at 3 weeks (*p* = 0.041) after TAC (Fig. 3d). Levels of TGF-β2 and TGF-β3 (Supplementary Fig. S2) were not different between WT and TLR2^−/−^ mice after TAC. These higher TGF-β1 levels at 2 and 3 weeks in WT hearts preceded the increase in interstitial fibrosis in WT hearts at 4 and 8 weeks after TAC (Fig. 2b).

### TLR2 deficiency reduced LV inflammatory cytokine/chemokine levels, but not inflammatory cell influx, following TAC

**Figure 4:**
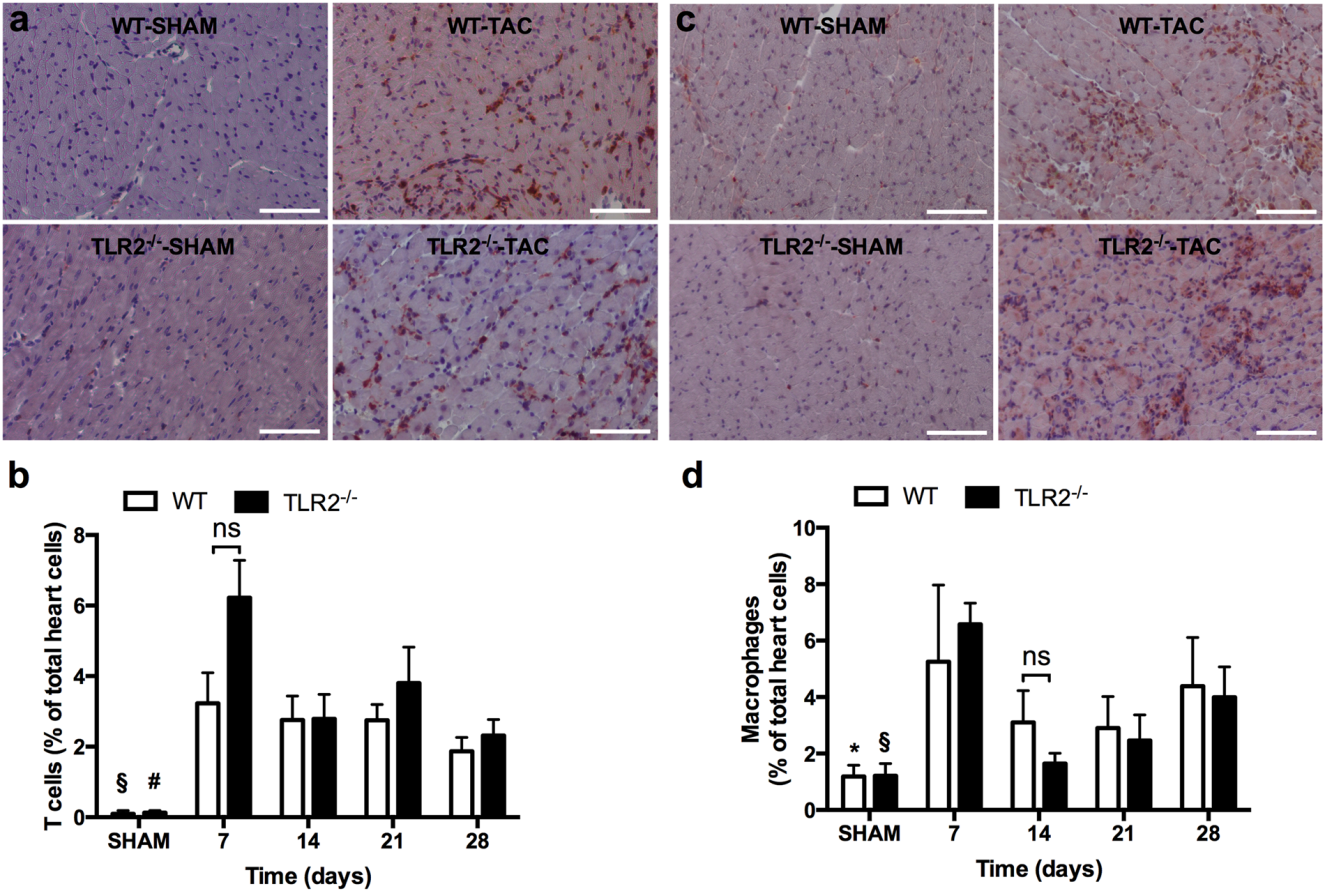
Influx of T cells and macrophages to the heart in response to pressure overload. LV sections were stained and quantified for CD3 positive T cells (**a**,**b**) and MAC3 positive macrophages (**c**,**d**) by immunohistochemistry. Representative sections stained for T cells (**a**; in brown) and macrophages (**c**; in brown) and the cell nuclei were counterstained in blue. Scale bars = 100 μm. (**b**,**d**) Quantification of T cells and macrophages at indicated timepoints after TAC. Bars represent mean ± SEM. Mouse hearts were extracted at indicated timepoints, n = 6–10 mice per genotype per timepoint. Mann Whitney U test was performed to determine the difference between groups; *p < 0.05, ^§^ *p* < 0.01 and ^#^ *p* < 0.001 indicate the differences between SHAM and 7 days after TAC for WT and TLR2^−/−^ mice, respectively; ns indicates not significant.

TAC induced the recruitment of inflammatory cells such as T cells and macrophages in the heart^17, 18^ (Fig. 4). Immunohistochemistry showed that a large number of CD3 positive T cells (Fig. 4a,b) and MAC3 positive macrophages (Fig. 4c,d) were recruited to the hearts in both WT and TLR2^−/−^ mice after TAC compared to the respective sham-operated animals. However, no differences in the increase of T cells or macrophages were observed between WT and TLR2^−/−^ mouse groups (Fig. 4b,d). To confirm these data, we also isolated cells from heart tissue at 1 week and 3 weeks after TAC for flow cytometric analysis (FACS). FACS showed that similar amount of inflammatory cells were recruited to the hearts of WT and TLR2^−/−^ mice in response to pressure overload (Supplementary Fig. S3).

Cytokine/chemokine levels were determined in heart tissue and plasma (Table 1). Lower levels of IL-1α, IL-2, IFN-γ, MCP-1 and MIP-1α protein were found in TLR2^−/−^ hearts after TAC but were not detectable in the plasma. Lower levels of plasma TNFα, IL-6, KC, Rantes, IL12p40, IL12p70, IL3, and G-CSF were found in TLR2^−/−^ mice compared to WT mice after TAC. The level of anti-inflammatory factor MIP-1β^19^ was higher in TLR2^−/−^ hearts.

**Table 1:** Level of cytokines and chemokines in the heart and plasma after TAC.

Protein	WT					TLR2^−/−^				
	Baseline	Day 7	Day 14	Day 21	Day 28	Baseline	Day 7	Day 14	Day 21	Day 28
**Cytokines and chemokines in the heart (pg/mg)**										
MCP-1	27 ± 7	183 ± 127	37 ± 2	39 ± 4	41 ± 7	20 ± 4	56 ± 7	29 ± 5	25 ± 2^§^	26 ± 2*
IL-1α	18 ± 5	22 ± 3	25 ± 1	36 ± 1	36 ± 7	24 ± 2	17 ± 3	20 ± 4	18 ± 3*	27 ± 6
IL-2	37 ± 14	32 ± 4	39 ± 3	64 ± 8	42 ± 8	27 ± 4	32 ± 8	22 ± 5*	26 ± 6^§^	16 ± 4*
IFN-γ	54 ± 17	26 ± 7	51 ± 3	70 ± 13	78 ± 33	54 ± 17	23 ± 5	45 ± 13	28 ± 5*	33 ± 7
MIP-1α	5 ± 1	50 ± 38	9 ± 1	8 ± 2	10 ± 2	3 ± 1	11 ± 2	14 ± 10	4 ± 0	5 ± 0*
MIP-1β	5 ± 1	18 ± 10	6 ± 1	6 ± 1	5 ± 0	6 ± 1	18 ± 3	13 ± 5	13 ± 3*	15 ± 3§
**Cytokines and chemokines in the plasma (pg/mL)**										
IL-3	29 ± 2	29 ± 2	30 ± 5	36 ± 1	27 ± 2	28 ± 4	23 ± 3	16 ± 2	24 ± 3§	21 ± 3
IL-12p40	97 ± 7	124 ± 11	122 ± 12	110 ± 10	100 ± 15	113 ± 10	87 ± 6*	79 ± 11*	99 ± 8	98 ± 7
IL-12p70	204 ± 20	195 ± 38	195 ± 38	246 ± 13	188 ± 17	189 ± 27	149 ± 20	101 ± 15*	153 ± 19^§^	135 ± 20
G-CSF	45 ± 4	46 ± 4	42 ± 4	59 ± 7	38 ± 2	41 ± 9	30 ± 3*	22 ± 3*	31 ± 3^#^	39 ± 7
KC	27 ± 3	41 ± 10	33 ± 4	38 ± 3	27 ± 3	25 ± 5	34 ± 5	18 ± 4*	26 ± 1^§^	34 ± 6
MIP-1β	38 ± 3	38 ± 4	34 ± 5	37 ± 3	30 ± 3	39 ± 3	42 ± 4	33 ± 4	41 ± 6	29 ± 4
RANTES	6 ± 1	7 ± 0	7 ± 1	7 ± 0	6 ± 1	5 ± 1	4 ± 0*	3 ± 1*	4 ± 0^§^	5 ± 0
TNF-α	427 ± 24	395 ± 21	393 ± 52	451 ± 27	333 ± 25	394 ± 29	422 ± 53	291 ± 36*	457 ± 67	267 ± 46
IL-6	19 ± 10	11 ± 1	8 ± 1	12 ± 1	8 ± 1	14 ± 3	9 ± 1	7 ± 1	8 ± 1*	8 ± 1

Mouse hearts and plasma were obtained at indicated timepoints for measurement of protein levels with multiplex assays. ^*^ *p* < 0.05, ^§^ *p *< 0.01, ^#^ *p* < 0.001, non-parametric test compared to WT mice at respective timepoints. N = 6–8 per timepoint per genotype of mice. MCP-1, monocyte chemoattractant protein-1; IL-1α, interleukin 1 alpha; IL-2, interleukin 2; IL-3, interleukin 3; IL-6, interleukin 6; IFN-γ, interferon gamma; MIP-1α, macrophage inflammatory protein 1 alpha; MIP-1β, macrophage inflammatory protein 1 beta; IL-12p40, interleukin-12 p40; IL-12p70, interleukin-12 p70; G-CSF, granulocyte-colony stimulating factor; KC, keratinocyte chemoattractant; RANTES, Regulated on Activation, Normal T Cell Expressed and Secreted, also known as chemokine (C-C motif) ligand 5 (CCL5); TNF-α, tumor necrosis factor-alpha.

### TLR2 deficiency on bone marrow-derived cells mediated cardiac protective effects following TAC

**Figure 5:**
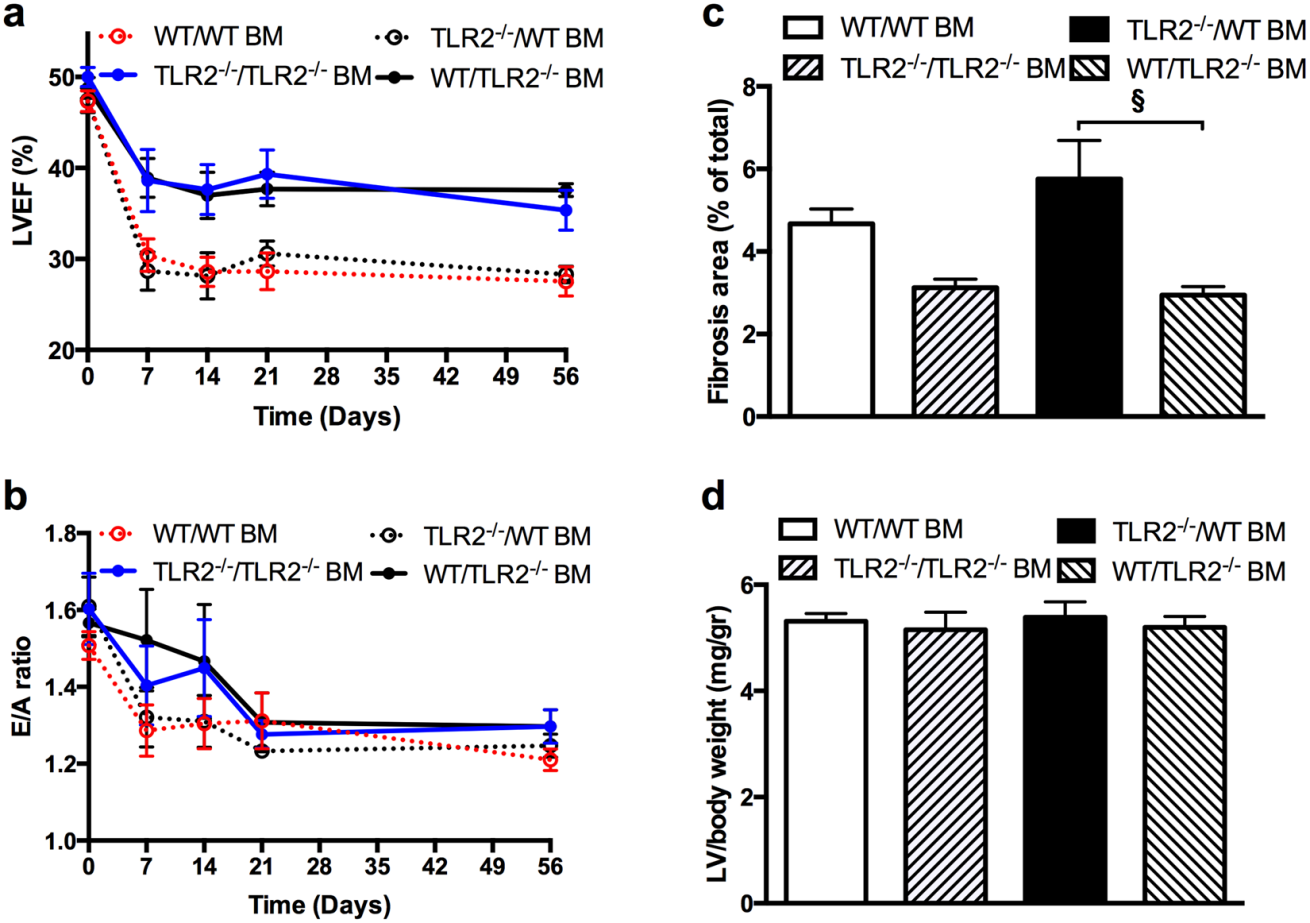
Cardiac function, fibrosis and hypertrophy after TAC in bone marrow chimaeric mice. (**a**) LVEF measured by echocardiography at 0 day (baseline), 7 days, 14 days, 21 days, and 56 days after TAC in 4 types of BM chimaeric mice consisting of WT mice with TLR2^−/−^ BM (WT/TLR2^−/−^ BM), TLR2^−/−^ mice with WT BM (TLR2^−/−^/WT BM), WT with WT BM (WT/WT BM) and TLR2 with TLR2 BM (TLR2^−/−^/TLR2^−/−^ BM) showing a large difference between the 4 types of BM transplantation (p < 0.001). (**b**) E/A ratio measured by echocardiography at 0 day, 7 days, 14 days, 21 days, and 56 days after TAC in the 4 types of BM chimaeric mice. (**c)** Fibrotic area as % of LV area at 8 weeks after TAC using Picrosirius Red staining in the 4 types of BM chimaeric mice. Fibrotic area was different between the 4 types of BM transplantation analyzed with Kruskal-Wallis test (p = 0.001). Difference in fibrotic area between WT/TLR2^−/−^ BM and TLR2^−/−^/WT BM was determined with Mann-Whitney U test; ^§^ *p* < 0.01. (**d**) Hypertrophy as measured by wet LV weight corrected for body weight in mg/g at 8 weeks after TAC in the 4 types of BM chimaeric mice. N = 6–10 per type of BM chimaeric mice.

Bone-marrow (BM) transplantation was performed to determine if preservation of cardiac function was dependent on TLR2 on BM-derived cells. Four groups of chimaeric mice were created: recipient WT mice with TLR2^−/−^ BM (WT/TLR2^−/−^ BM mice), recipient WT mice with WT BM (WT/WT BM mice), recipient TLR2^−/−^ mice with TLR2^−/−^ BM (TLR2^−/−^/TLR2^−/−^ BM mice) and recipient TLR2^−/−^ mice with WT BM (TLR2^−/−^/WT BM mice). BM chimaerization was confirmed by flow cytometry 6 weeks after transplantation (<5% TLR2^+/+^ leukocytes in WT/TLR2^−/−^ BM mice). As shown in Fig. 5a, LVEF was different among the four groups of BM chimaeric mice (*p* < 0.0001). Post hoc analysis showed that LVEF in WT/WT BM mice and TLR2^−/−^/WT BM mice was lower than that in WT/TLR2^−/−^ BM mice and TLR2^−/−^/TLR2^−/−^ BM mice (*p* = 0.002). LVEF in WT/TLR2^−/−^ BM mice was higher than that in TLR2^−/−^/WT BM mice at all the timepoints after TAC (*p* < 0.05). No differences in LVEF were found between WT/WT BM mice and TLR2^−/−^/WT BM mice, neither between WT/TLR2^−/−^ BM mice and TLR2^−/−^/TLR2^−/−^ BM mice. In contrast to LVEF, no significant difference was detected in E/A ratio among the four types of BM chimaeric mice (*p* = 0.246, Fig. 5b). To investigate if radiation differentially affected E/A ratio in TLR2^−/−^ and WT mice, E/A ratio at baseline (before TAC) in TLR2^−/−^ mice that were not radiated was compared to that in radiated TLR2^−/−^ mice received TLR2^−/−^ BM (TLR2^−/−^/TLR2^−/−^ BM mice) 6 weeks after BM transplantation. This was also done for WT mice. The results revealed no difference in E/A ratio in TLR2^−/−^ mice (1.71 ± 0.04 for TLR2^−/−^ mice before radiation versus 1.61 ± 0.09 for TLR2^−/−^/TLR2^−/−^ BM mice, *p* = 0.476). The E/A ratio in WT mice, however, did differ before and after radiation (1.71 ± 0.03 for WT mice before radiation versus 1.52 ± 0.04 for WT/WT BM mice, *p* = 0.006).

To examine whether cardiac fibrosis was attributed to TLR2 on the BM-derived leukocytes, collagen deposition in LV tissue from BM chimaeric mice was analyzed with Picrosirius Red staining. As shown in Fig. 5c, interstitial fibrosis at 8 weeks after TAC was different among the 4 types of BM chimaeric mice (*p* = 0.003). Among the groups, fibrosis was lower in WT/TLR2^−/−^ BM mice compared to TLR2^−/−^/WT BM mice (*p* = 0.001) and WT/WT BM mice (*p* = 0.021) but similar compared to TLR2^−/−^/ TLR2^−/−^ mice (*p* = 0.841). LV hypertrophy at 8 weeks after TAC, as determined by LV wet weight corrected for body weight, did not differ among the 4 types of BM chimaeric mice (Fig. 5d, *p* = 0.895).

## Discussion

The role of TLR2 in cardiac remodeling and dysfunction following ischemic cardiac injuries has been established^7, 8, 20, 21^; however, its role in the setting of sustained pressure overload remains unclear. We now provide a longitudinal study up to 8 weeks after TAC focusing on the temporal changes in cardiac function, hypertrophy, fibrosis and inflammation. Our data demonstrate that TLR2 deficiency preserves both cardiac systolic and diastolic function via reduction of fibrosis and inflammation but not hypertrophy induced by pressure overload. The effects of TLR2 on pressure overload induced cardiac dysfunction are attributed to TLR2 on the BM-derived leukocytes.

Cardiac hypertrophy is a major (mal-)adaptive response to pressure overload as well as an important risk factor for heart failure in hypertension^22^. The role of TLR2 in cardiac hypertrophy is however conflicting in the literature. Higashikuni *et al*. showed a lower heart weight in TLR2^−/−^ mice measured at 14 days and 28 days after TAC^12^. In contrast, another study found a higher heart weight in TLR2^−/−^ mice compared to WT at 14 and 28 days but no difference was detected in left ventricle weight between WT and TLR2^−/−^ mice^13^. We found in this study that hypertrophy, as evidenced by the increase in LV weight and mRNA levels of two hypertrophic markers ANP and BNP, occurred in both WT and TLR2^−/−^ mice to a similar extent from week 1 up to week 8 in response to sustained pressure overload (Fig. 1a and Supplementary Fig. S1), showing that TLR2 is not involved in hypertrophy. The discrepancies among the three studies may be explained by a few reasons. Higashikuni *et al*.^12^ used a milder 25 gauge needle that is in line with no difference in heart weight between TLR2^−/−^ and sham at 28 days. We and Bualeong *et al*.^13^ achieved more severe aortic constriction using a 27 gauge needle, and both studies did not find a difference in left ventricular weight suggesting that the role of TLR2 probably depends on the severity of pressure overload. Furthermore, both studies found that lung weight after TAC did not differ between TLR2^−/−^ and WT mice. Besides clear differences in procedure, the role of the mouse origin is unclear^13^ and in all these studies mice are on a C57BL/6 background.

Severe pressure overload leads to cardiac dysfunction as indicated by decreased LVEF^23–26^. In line with this, we observed a quick and strong reduction of LVEF at 7 days following severe TAC with 27 gauge needle in WT mice (*p* = 0.003) (Fig. 1c). LVEF was higher in TLR2^−/−^ mice compared to WT mice from 1 week up to 8 weeks after TAC. This is in contrast with Higashikuni *et al*.^12^ who found that LVEF in TLR2^−/−^ was lower than WT at 14 and 28 days after TAC. In this earlier study, however, LVEF in WT mice at 14 days after TAC was not different from sham, in contrast to a decrease in LVEF at the same time point after TAC in C56Bl/6 mice described in other studies^23–26^. Once again, this discrepancy may be due to differences in the severity of the imposed pressure overload using the 25 gauge versus 27 gauge needles.

Left ventricular diastolic dysfunction occurs in hypertensive heart disease in both human and animals^15, 27^. The mitral E/A ratio, a parameter of LV diastolic function, has not previously been measured in TLR2^−/−^ mice. We showed for the first time that after TAC, mitral E/A ratio was preserved in association with reduced LV fibrosis in TLR2^−/−^ hearts. Together with higher fibrosis in WT hearts, Col1a1 and Col3a1 mRNA levels as well as TGFβ1 protein levels involved in collagen synthesis were higher in WT hearts, while MMP levels did not differ. TGFβ1 protein levels were higher in WT just before fibrosis increased. Collectively, these findings suggest that reduced LV fibrosis in TLR2 deficient hearts following TAC was related to decreased collagen synthesis rather than increased collagen breakdown. This is in agreement with the lower mRNA levels of Col3a1 and TGFβ1 in TLR2^−/−^ hearts after TAC described in an earlier study^12^. A lower level of interstitial fibrosis in TLR2^−/−^ hearts has also been described in doxorubicin-induced cardiomyopathy^10^ and in response to angiotensin perfusion^11^.

In the pressure-overloaded heart, interstitial fibrosis causes cardiac dysfunction and inflammation is believed one of the main stimulators of fibrosis^14^. In line with this, we observed a large number of T cells and macrophages recruited to the heart after TAC as previously reported^17, 18^ as well as an elevation of cytokines/chemokines in both plasma and heart tissue in response to pressure overload, preceding cardiac fibrosis (Figs 2 and 4 and Table 1). In contrast to a reduced influx of inflammatory cells after myocardial ischemia reperfusion injury^8^, TLR2 deficiency did not reduce inflammatory cell influx into the pressure-overloaded heart compared to WT mice. TLR2 deficiency, however, reduced cytokines/chemokines levels in both plasma and heart tissue after TAC. This suggests that cardiac inflammation in pressure overload is regulated by TLR2 via production of cyto/chemokines rather than recruitment of inflammatory cells to the heart. As a result, the reduced production of cytokines/chemokines in TLR2^−/−^ mice may subsequently lead to less activation of profibrotic pathways in myofibroblasts and therefore decelerate fibrosis.

Our BM transplantation experiments showed that it was TLR2 deficiency on BM-derived cells rather than the parenchymal cells (such as cardiomyocytes and endothelial cells) responsible for the preservation of LVEF and reduction of fibrosis in TLR2^−/−^ mice subjected to sustained pressure overload. This is in agreement with a previous study showing that after ischemia/reperfusion the infarct size was determined by TLR2 on BM-derived cells^8^. Adverse remodeling after myocardial infarction has also been shown to be dependent on bone marrow TLR4^28^ or NFkB-p50^29^. In contrast to our findings, Higashikuni *et al*.^12^ concluded that lack of TLR2 on the parenchymal heart cells but not the BM-derived cells was involved in regulation of cardiac function. Reasons for this discrepancy are unclear but may involve differences in methodology or mouse strain.

Finally, our BM transplantation experiments revealed the surprising finding that TLR2 deficiency might protect hearts from radiation-induced diastolic dysfunction. Radiation is known to induce fibrosis and diastolic dysfunction in the heart^30^. We therefore hypothesized that TLR2^−/−^ mice may have reduced radiation-induced fibrosis and thereby preserved diastolic dysfunction. Indeed we found that following radiation alone without TAC, WT mice had lower E/A ratio whereas TLR2^−/−^ mice had preserved E/A ratio. This differential response to radiation in TLR2^−/−^ versus WT mice will confound the interpretation of E/A ratios following BM transplantation and TAC. Still, the implication of this unexpected finding for the role of TLR2 in radiation-induced injury deserves further study.

In summary, our data show that TLR2 on BM-derived leukocytes is involved in the response of the heart to sustained pressure overload. TLR2 deficiency does not affect hypertrophy but reduces fibrosis and protects from cardiac systolic and diastolic dysfunction. Targeting leukocytic TLR2 may provide a novel therapeutic target to prevent cardiac dysfunction and heart failure due to chronic hypertension.

## Methods

### Animals and ethics approval

C57BL/6J (wild type, WT) and TLR2^−/−^ mice (Stock No: 004650) were purchased from Jackson Laboratory and were maintained under a 12/12‐hour light‐dark cycle (lights on at 7 AM, lights off at 7 PM) at the Comparative Medicine Animal Vivarium at National University of Singapore. The mice received standard diet and water ad libitum. Genotyping was routinely performed with the TLR2 specific primers as recommended by the Jackson Laboratory (Supplementary Fig. S4)^31, 32^: WT forward 5′-ACGAGCAAGATCAACAGGAGA-3′;mutant forward 5′-GGGCCAGCTCATTCCTCCCAC-3′; common reverse (for both genotypes) 5′-CTTCCTGAATTTGTCCAGTACA-3′. Male WT or TLR2^−/−^ mice (10–12 weeks old; 20–25 g) were used for all experiments. Animal numbers used for each experiment were indicated in the table and figure legends. All the procedures involving animal handling were performed with prior approval and in accordance with the protocols and guidelines of the Institutional Animal Care and Use Committee (IACUC), National University of Singapore.

### Generation of pressure overload in mice

Mice were anesthetized with a mixture of 0.5 mg/kg medetomidine (Pfizer Animal Health, Exton, PA, USA), 5.0 mg/kg Dormicum (Sciencelab.com, Inc., Texas, USA) and 0.05 mg/kg Fentanyl (Pfizer Pharmaceuticals Group, New York, USA) via intra-peritoneal injection, intubated and ventilated with a rodent ventilator (Harvard Apparatus). Transverse aortic constriction (TAC) was performed as previously described^25^. Briefly, the transverse aortic arch was exposed by a median sternotomy and bonded against a blunt 27-gauge needle with a 7–0 suture followed by prompt removal of the needle. Sham operated mice underwent the same procedure without aortic binding. The mice were recovered from anesthesia by subcutaneous injection of 2.5 mg/kg Atipamezole (Pfizer Animal Health, Exton, PA, USA) and 0.5 mg/kg Flumazenil (Sagent Pharmaceuticals, Illinois, USA) followed by 0.1 mg/kg Temgesic (Hospira Inc., Illinois, USA) for analgesia. Sustained pressure overload was induced with less than 10% mortality in both WT and TLR2^−/−^ mice during 8 weeks follow-up. Mice with ratio of right to left carotid artery flow between 6–8 at both week 3 and week 8 post-TAC were included for this study.

### Bone marrow transplantation

Chimeric mice were generated as previously described^8^ to study the contribution of TLR2 expression on circulating cells and parenchymal cells to pressure overload-induced heart failure. Bone marrow (BM) cells were collected from WT and TLR2^−/−^ mice by flushing humeri, femurs and tibiae with RPMI-1640 medium. Recipient mice received 5 × 10^6^ BM cells after receiving a single dose of 7 Gy radiation from a Biobeam 8000 (^137^Cs source) irradiator (Gamma-Service Medical GmbH, Leipzig, Germany). Mice were allowed to recover for 6 weeks to ensure stable engraftment of the donor BM cells. Hereafter, chimerization was confirmed by flow cytometry analysis of TLR2 expression on peripheral blood cells (rat-anti-mouse TLR2 monoclonal antibody conjugated with FITC, eBioscience Inc., San Diego, CA, USA) with CyAn ADP Analyzer (Beckman Coulter, Indianapolis, IN, USA). Recipient WT mice with TLR2^−/−^ BM were referred to as WT/TLR2^−/−^ BM mice, and recipient TLR2^−/−^ mice with WT BM were called TLR2^−/−^/WT BM mice.

### Quantitative Real‐Time‐PCR analysis

Left ventricles were minced and grinded in liquid nitrogen. Total mRNA was extracted from mouse heart tissue with RNeasy Mini Kit (Qiagen, Hilden, Germany) following manufacturer’s instruction. cDNA was synthesized with 250 ng total mRNA using QuantiTect-Reverse-Transcription Kit (Qiagen). qPCR was performed in triplicate with iTaq Universal SYBR Green Supermix (Bio-Rad, Hercules, CA, USA) and measured in CFX96 Touch™ Real-Time PCR Detection System (Bio-Rad). GAPDH was used as an internal control. The primers used for qRT-PCR included: GAPDH, 5′-GTGGAGTCATACTGGAACATGTAG-3′ (forward) and 5′-AATGGTGAAGGTCGGTGTG-3′ (reverse); Col1a1, 5′-TCAAGGTCTACTGCAACATGG-3′ (forward) and 5′-AATCCATCGGTCATGCTCTCT-3′ (reverse); Col3a1, 5′-GATGCCATTAGAGCCACGTT-3′ (forward) and 5′-AAGAGTGGTGACAGAGGAGAA-3′ (reverse); ANP, 5′-AGGTGGTCTAGCAGGTTCT-3′ (forward) and 5′-CTTCCTCGTCTTGGCCTTT-3′ (reverse); BNP, 5′-CTTTTCTCTTATCAGCTCCAGCA-3′ (forward) and 5′-CTGCTTTTCCTTTATCTGTCACC-3′ (reverse).

### Quantitative measurement of cytokines and chemokines

Concentrations of cytokines and chemokines in heart tissue and plasma were measured with the Bio-Plex Pro Mouse Cytokine 23-Plex immunoassay and the Pro TGF-β 3-Plex Immunoassay (Bio-Rad) on a Bio-Plex 200 multiplex suspension array system (Bio-Rad) according to the manufacturer’s protocol. Snap-frozen left ventricles were minced and proteins were isolated with Bio-Plex™ Cell Lysis Kit (Bio-Rad). 1.5 mg of protein per sample was loaded and concentrations of analytes were expressed as pg/mg protein. Undiluted plasma samples were used for multiplex assay and concentrations of analytes were expressed as pg/mL.

### MMP-2 and MMP-9 activity assays

Total protein was extracted from mouse heart tissue with Tris-HCI buffer (50 mM, pH7.8) containing 0.1% Tween 20. The tissue was homogenized in the buffer followed by centrifugation at 10,000 g for 15 min. Supernatant was collected and used for analyzing MMP-2 and MMP-9 activity with respective kits (QuickZyme Biosciences, Leiden, The Netherlands). 3 mg of protein per sample was loaded and concentrations of active MMPs were expressed as pg/mg protein.

### Immunohistochemistry

Isolated mouse left ventricles were fixed with 4% formalin and embedded in paraffin. Tissue sections (5 μm) were stained for CD3 (T cells; rabbit anti-human CD3, clone F7.2.38, Dako, Glostrup, Denmark) and MAC-3 (macrophages; rat anti-mouse MAC-3, clone M3/84, BD Biosciences, Heidelberg, Germany). Sections were incubated with the primary antibodies at 4 °C overnight followed by incubation with goat anti-rat (Life technologies, Singapore) or goat anti-rabbit (Abcam, Cambridge, UK) secondary antibodies conjugated to horseradish peroxidase (HRP) at room temperature for 1 hour. The NovaRED Peroxidase (HRP) Substrate kit was used to visualize the staining according to the manufacturer’s instruction (Vector Laboratories, Burlingame, Ca, USA). All sections were counterstained with haematoxylin to visualize cell nuclei. Staining was imaged under a Nikon Eclipse Ti inverted microscope (Nikon Instruments Inc., Tokyo, Japan) and analyzed by NIS-Element AR Analysis software 4.5 version (Nikon Instruments Inc.). T cells and macrophages were quantitated in the whole ventricle area by automatic detection and the results were presented as percentage of total heart cells. Interstitial fibrosis was quantified by Picrosirius Red staining of collagen and expressed as percentage of total tissue area. Three whole ventricle sections were used to quantify inflammatory cells or fibrosis for each mouse heart.

### Cardiac function assessment

Cardiac function was assessed with a high frequency ultrasound system Vevo^®^ 2100 (Visualsonics, Toronto, Canada) and analyzed with Vevo^®^ 2100 software, version 1.7.0. Echocardiography was performed on mice under general anesthesia (1–1.5% isoflurane, Baxter, Singapore) at indicated time points. Body temperature was monitored with a rectal probe and maintained at 36–37 °C. Volumes and functional parameters were measured and analyzed by a blinded researcher.

### Statistical analysis

Comparisons between groups in time were performed using a General Linear model (GLM) for multivariate analysis or GLM for repeated measurements with LSD post hoc testing. Mann Whitney U test was used to determine differences between groups at individual timepoints. Related-Samples Wilcoxon Signed Rank test was used to determine difference between time points. Values were reported as mean ± SEM. *P*-value ≤ 0.05 was considered statistically significant. Data were analyzed with SPSS software (IBM^®^ SPSS^®^ Statistics version 22.0).

### Data availability

All data generated or analysed during this study are included in this published article and its Supplementary Information files.

## Electronic supplementary material

Supplementary Information
