# Supplementary material for: Leukocytic Toll-Like Receptor 2 Deficiency Preserves Cardiac Function And Reduces Fibrosis In Sustained Pressure Overload
Source: Sci Rep. 2017 Aug 23;7:9193. doi: 10.1038/s41598-017-09451-3 (PMC5569043; doi:10.1038/s41598-017-09451-3)
Supplement: Supplementary file 1 — Supplementary Information [file 41598_2017_9451_MOESM1_ESM.pdf]

Leukocytic Toll-Like Receptor 2 Deficiency Preserves Cardiac Function And Reduces Fibrosis In Sustained Pressure Overload

Jiong-Wei Wang[1], Magda S.C. Fontes[2], Xiaoyuan Wang[1], Suet Yen Chong[1], Elise L. Kessler[2], Ya-Nan Zhang[1], Judith J. de Haan[3], Fatih Arslan[3], Saskia C.A. de Jager[3,4], Leo Timmers[3], Toon A.B. van Veen[2], Carolyn S.P. Lam[5,*], Dominique P.V. de Kleijn[1,3,6,7,*]

[1]Department of Surgery, YLL School of Medicine, National University of Singapore & Cardiovascular Research Institute (CVRI), National University Heart Centre Singapore (NUHCS) and National University Healthy System (NUHS), Singapore

[2]Department of Medical Physiology, Division of Heart & Lungs, University Medical Center Utrecht, Utrecht, The Netherlands

[3]Department of Cardiology, University Medical Center Utrecht, Utrecht, The Netherlands

[4]Laboratory of Translational Immunology, University Medical Center Utrecht, Utrecht, The Netherlands

[5]National Heart Centre Singapore, Duke-NUS Graduate Medical School, Singapore & Cardiology, University Medical Center, Groningen, the Netherlands.

[6]Department of Vascular Surgery, University Medical Center Utrecht, Utrecht, the Netherlands

[7]Netherlands Heart Institute, Utrecht, the Netherlands

SUPPLEMENTARY DATA

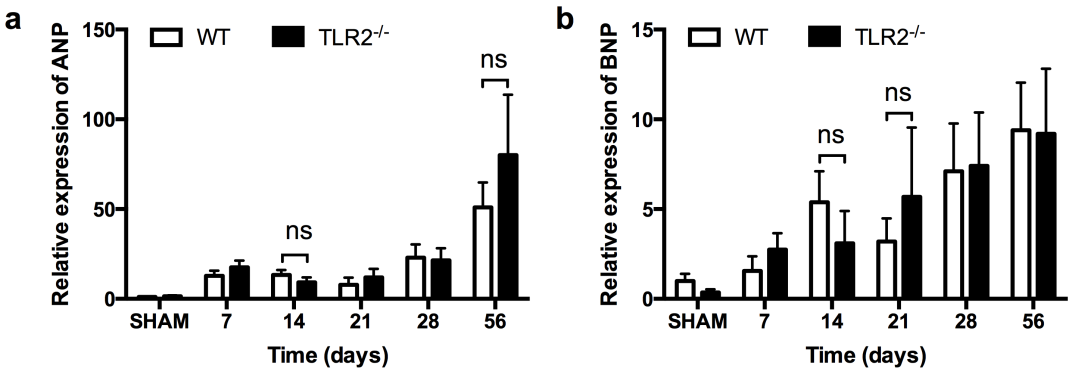

Supplementary Figure S1. Expression of cardiac hypertrophic markers. mRNA levels of atrial natriuretic peptide (ANP) and brain natriuretic peptide (BNP) in the heart were determined at indicated timepoints after transverse aortic constriction

(TAC). Relative expression of ANP (a) and BNP (b) was quantified by qRT-PCR and normalized to wild type (WT) SHAM animals. GAPDH was used as an internal control. Bars represent mean±SEM. Mouse hearts were harvested at indicated timepoints, n=5-8 mice per group per timepoint. Mann Whitney U test was performed to determine the difference between groups at individual timepoints; ns, not significant.

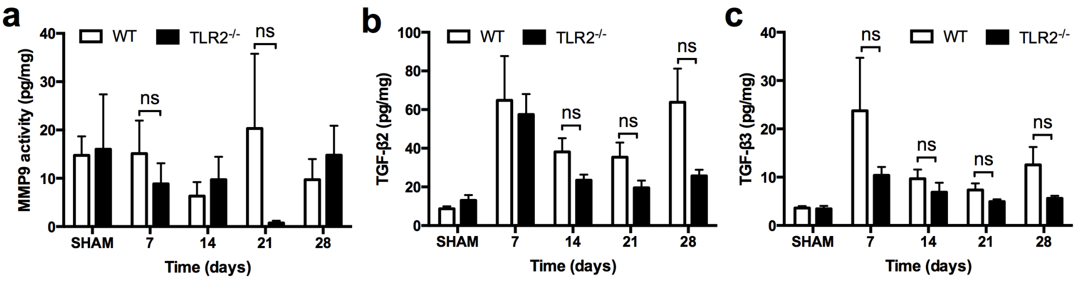

Supplementary Figure S2. MMP9 activity, TGF-β2 and TGF-β3 protein levels in heart tissue. (a) Activity of MMP9 was determined in mouse hearts extracted from sham-operated mice (SHAM) or mice subjected to TAC at 7 days, 14 days, 21 days and 28 days. (b-c) Protein levels of TGF-β2 and TGF-β3 in heart tissue were determined by multiplex assay at indicated timepoints. Bars represent mean±SEM. N=6-8 mice per genotype per timepoint. Mann Whitney U test was performed to determine the difference between groups at individual timepoints; ns, not significant.

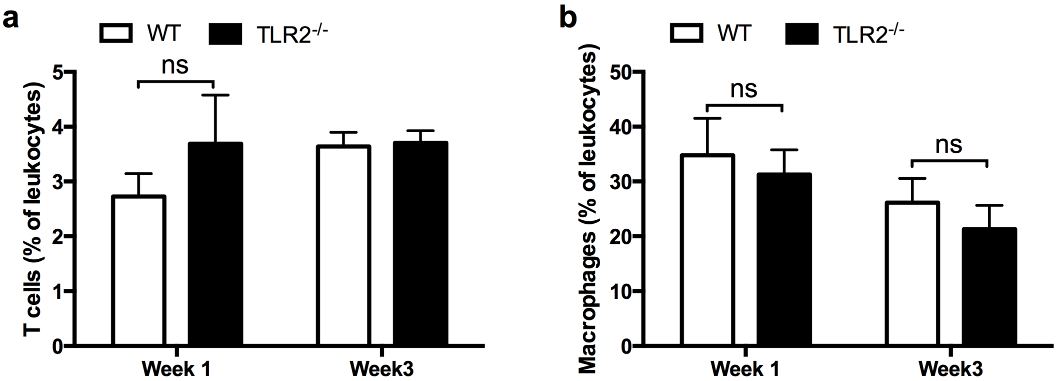

Supplementary Figure S3. Recruitment of inflammatory cells to the heart after sustained pressure overload. Extracted left ventricles were minced into small pieces (around 1 mm^2) and digested at 37°C for 20 min under gentle agitation in Dulbecco's Phosphate Buffered Saline conatining 1000 U/ml DNase I (Sigma-Aldrich), 2.6 U/ml Liberase TL (Roche Diagnostics) and 10 mM HEPES. The dissociated cells were filtered with a 40 µm cell strainer (BD Biosciences). For flow cytometry, cells were resuspended and stained with PE-CF594 rat anti-mouse CD45

(BD Biosciences) for leukocytes, Alexa Fluor® 700 rat anti-mouse CD4 (eBiosciences) for T cells, PE-Cyanine7 rat anti-mouse F4/80 (eBiosciences) for macrophages. Bars represent mean±SEM. N=4 per group for mice sacrificed at 1 week after TAC; n=5 for WT and n=4 for TLR2$^{-/-}$ mice sacrificed at 3 weeks after TAC; ns, not significant, two-way ANOVA with Bonferroni post hoc test.

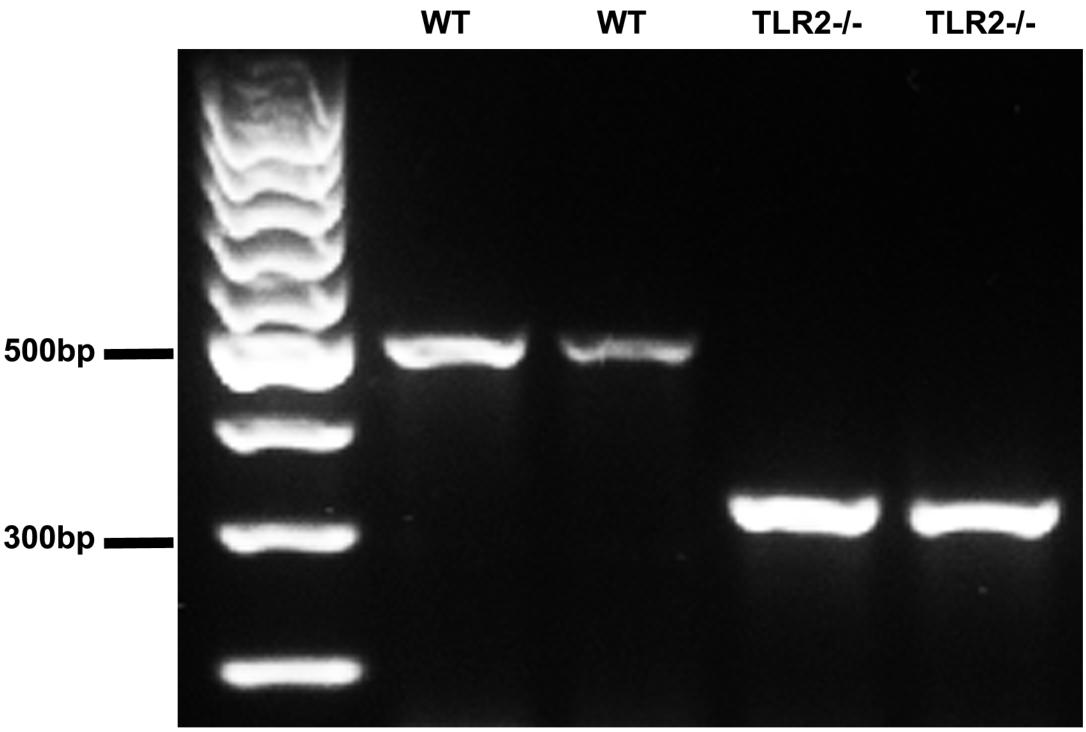

Supplementary Figure S4. Mouse genotyping. Mouse genomic DNA was extracted from the mouse ear biopsy. PCR was performed using DreamTaq Green PCR Master Mix (Thermo Fisher Scientific) according to the manufacture's instruction with a PCR thermal cycler (T100 Thermal Cycler, Bio-Rad). The yielded PCR products (wild type 499bp and mutant 334bp) were separated by 2% agarose gel electrophoresis (120V for 1 hour) and visualized with ChemiDoc Gel Imaging System (Bio-Rad). GeneRuler 100bp DNA Ladder (Thermo Fisher Scientific) was used as a molecular size reference. WT, wild type mice; TLR2$^{-/-}$, TLR2 deficient mice.
